# *N*-(2,5-Dimethyl­phen­yl)-2-methyl­benzamide

**DOI:** 10.1107/S160053681000886X

**Published:** 2010-03-13

**Authors:** B. Thimme Gowda, Miroslav Tokarčík, Jozef Kožíšek, Vinola Zeena Rodrigues, Hartmut Fuess

**Affiliations:** a Department of Chemistry, Mangalore University, Mangalagangotri 574 199, Mangalore, India; b Faculty of Chemical and Food Technology, Slovak Technical University, Radlinského 9, SK-812 37 Bratislava, Slovak Republic; c Institute of Materials Science, Darmstadt University of Technology, Petersenstrasse 23, D-64287 Darmstadt, Germany

## Abstract

In the title compound, C_16_H_17_NO, the two aromatic rings are almost coplanar, making a dihedral angle of 1.9 (2)°. The amide group makes dihedral angles of 48.0 (3) and 48.6 (3)° with the 2-methyl­phenyl and the 2,5-dimethyl­phenyl rings, respectively. Inter­molecular N—H⋯O hydrogen bonds link the mol­ecules into chains running along the *a* axis of the crystal.

## Related literature

For related structures, see Gowda, Foro *et al.* (2008**a* ▶,b* ▶); Gowda, Tokarčík *et al.* (2009 ▶).
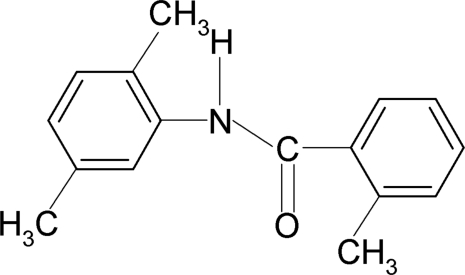


## Experimental

### 

#### Crystal data

C_16_H_17_NO
*M* *_r_* = 239.31
Orthorhombic, 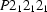

*a* = 4.90104 (10) Å
*b* = 5.85657 (16) Å
*c* = 45.8291 (12) Å
*V* = 1315.45 (6) Å^3^
*Z* = 4
Mo *K*α radiation
μ = 0.08 mm^−1^
*T* = 295 K
0.54 × 0.35 × 0.09 mm

#### Data collection

Oxford Diffraction Xcalibur Ruby Gemini diffractometer
Absorption correction: multi-scan (*CrysAlis PRO*; Oxford Diffraction, 2009 ▶) *T*_min_ = 0.957, *T*_max_ = 0.990
22131 measured reflections
1414 independent reflections
1338 reflections with *I* > 2σ(*I*)
*R*_int_ = 0.037

#### Refinement

*R*[*F* ^2^ > 2σ(*F* ^2^)] = 0.044
*wR*(*F* ^2^) = 0.097
*S* = 1.20
1414 reflections
165 parameters
H-atom parameters constrained
Δρ_max_ = 0.12 e Å^−3^
Δρ_min_ = −0.13 e Å^−3^

### 

Data collection: *CrysAlis PRO* (Oxford Diffraction, 2009 ▶); cell refinement: *CrysAlis PRO*; data reduction: *CrysAlis PRO*; program(s) used to solve structure: *SHELXS97* (Sheldrick, 2008 ▶); program(s) used to refine structure: *SHELXL97* (Sheldrick, 2008 ▶); molecular graphics: *ORTEP-3* (Farrugia, 1997 ▶) and *DIAMOND* (Brandenburg, 2002 ▶); software used to prepare material for publication: *SHELXL97*, *PLATON* (Spek, 2009 ▶) and *WinGX* (Farrugia, 1999 ▶).

## Supplementary Material

Crystal structure: contains datablocks I, global. DOI: 10.1107/S160053681000886X/dn2545sup1.cif

Structure factors: contains datablocks I. DOI: 10.1107/S160053681000886X/dn2545Isup2.hkl

Additional supplementary materials:  crystallographic information; 3D view; checkCIF report

## Figures and Tables

**Table 1:** Hydrogen-bond geometry (Å, °)

*D*—H⋯*A*	*D*—H	H⋯*A*	*D*⋯*A*	*D*—H⋯*A*
N1—H1*N*⋯O1^i^	0.86	2.05	2.899 (3)	172

Symmetry code: (i) 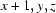.

**Fig. 1.**
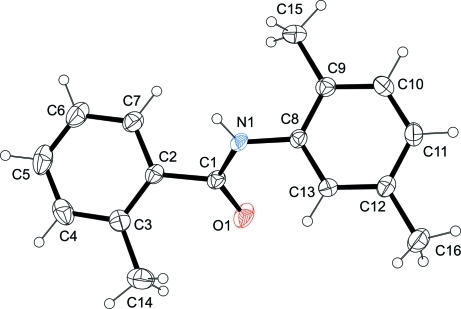
Molecular structure of the title compound showing the atom labelling scheme. Displacement ellipsoids are drawn at the 30% probability level and H atoms are represented as small spheres of arbitrary radii.

**Fig. 2.**
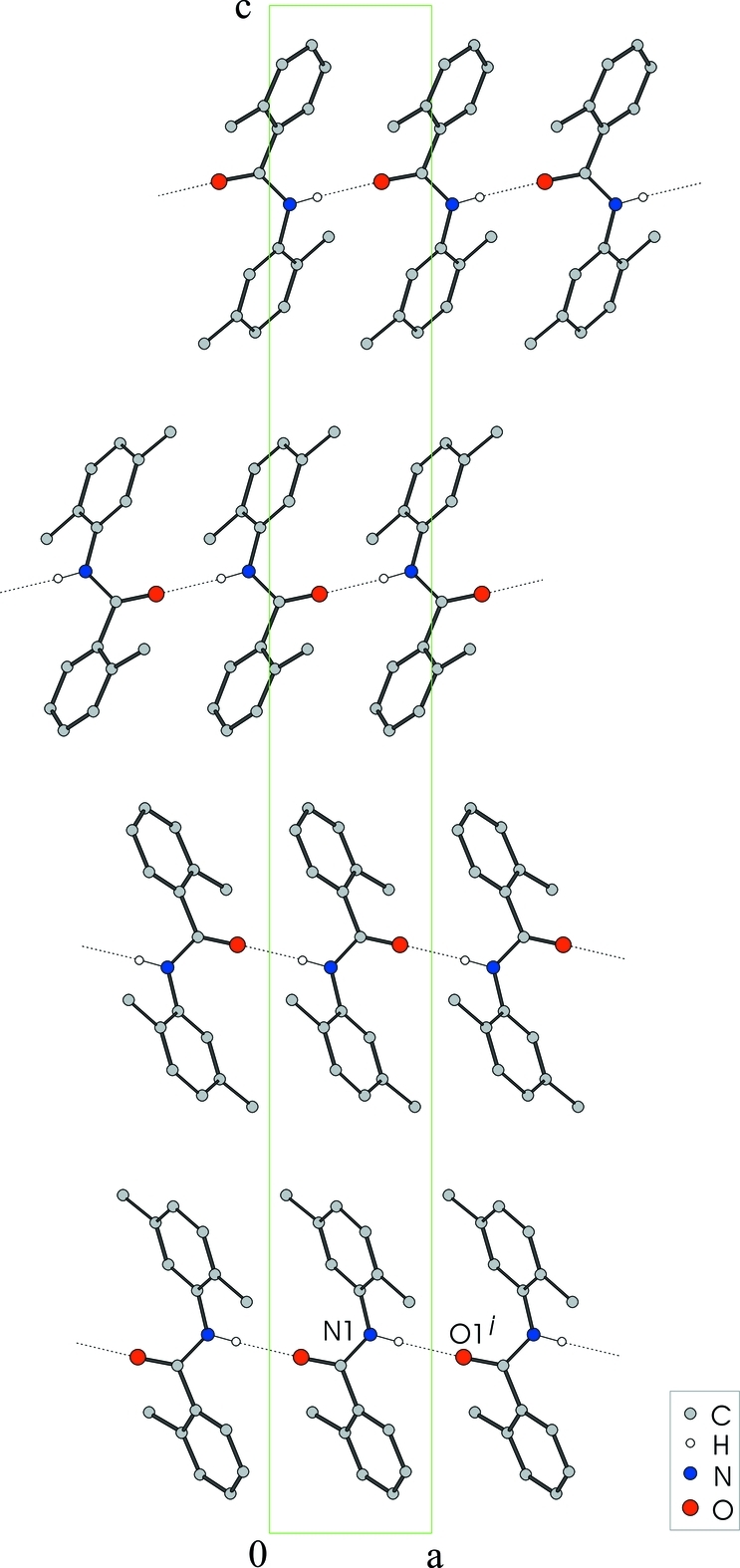
Molecular packing of the title compound with hydrogen bonds shown as dashed lines. H atoms not involved in hydrogen bonding have been omitted. [Symmetry code: (i) x + 1, y, z].

## supplementary crystallographic information

### Comment

As a part of our efforts to explore the effect of the substituents on the structures of benzanilides (Gowda, Foro *et al.*, 2008*a,b*; Gowda, Tokarčík *et al.*, 2009), in the present work, the structure of 2-methyl-*N*-(2,5-dimethylphenyl)benzamide (I) has been determined.

In the structure of (I) (Fig. 1), the N—H and C=O groups are in antiperiplanar conformation. This conformation is similar to those already observed, *e. g.* in 2-methyl-*N*-(phenyl)benzamide (II) (Gowda, Foro *et al.*, 2008*a*), 2-methyl-*N*-(2,6-dimethylphenyl)- benzamide (III) (Gowda, Foro *et al.*, 2008*b*) and in 2-methyl-*N*-(2,4-dimethylphenyl)benzamide (IV) (Gowda, Tokarčík *et al.*, 2009). Further in (I), the conformation of the C=O group to the methyl substituent in the 2-methylphenyl ring is *syn*. This conformation is similar to those observed in (II) and (IV). The bond parameters in (I) are similar to those in (II), (III) and (IV) and other benzanilides (Gowda, Foro *et al.*, 2008*a,b*; Gowda, Tokarčík *et al.*, 2009).

The two aromatic rings are almost coplanar, with the dihedral angle of 1.9 (2)°. The amido group makes dihedral angles of 48.0 (3)° and 48.6 (3)° with the 2-methylphenyl and the 2,5-dimethylphenyl rings, respectively. In the crystal structure, the intermolecular N–H···O hydrogen bonds (Table 1) link the molecules into chains running along the *a*-axis of the crystal (Fig. 2).

### Experimental

The title compound was prepared according to the method described by Gowda, Foro *et al.* (2008*b*). The purity of the compound was checked by determining its melting point. It was characterized by recording its infrared and NMR spectra. Block-like colourless single crystals of the title compound were obtained by slow evaporation from an ethanol solution (0.5 g in about 30 ml of ethanol) at room temperature.

### Refinement

All hydrogen atoms were positioned with idealized geometry using a riding model with C–H = 0.93 Å or 0.96 Å and N–H = 0.86 Å. The *U*_iso_(H) values were set at 1.2*U*_eq_(C-aromatic, N) and 1.5*U*_eq_(*C*-methyl). The C16 methyl group exhibits orientational disorder in the positions of H atoms. In the last cycles of refinement, all H atoms were treated as riding on their parent atoms.

The two sets of methyl hydrogen atoms were refined with occupancies 0.74 (4) and 0.26 (4). In the absence of significant anomalous scattering, the absolute structure could not be reliably determined and then the Friedel pairs were merged and any references to the Flack parameter were removed.

### Crystal data

**Table:**

C_16_H_17_NO	*F*(000) = 512
*M**_r_* = 239.31	*D*_x_ = 1.208 Mg m^−^^3^
Orthorhombic, *P*2_1_2_1_2_1_	Mo *K*α radiation, λ = 0.71073 Å
Hall symbol: P 2ac 2ab	Cell parameters from 11414 reflections
*a* = 4.90104 (10) Å	θ = 1.8–29.5°
*b* = 5.85657 (16) Å	µ = 0.08 mm^−^^1^
*c* = 45.8291 (12) Å	*T* = 295 K
*V* = 1315.45 (6) Å^3^	Block, colourless
*Z* = 4	0.54 × 0.35 × 0.09 mm

### Data collection

**Table:**

Oxford Diffraction Xcalibur Ruby Gemini diffractometer	1414 independent reflections
graphite	1338 reflections with *I* > 2σ(*I*)
Detector resolution: 10.434 pixels mm^-1^	*R*_int_ = 0.037
ω scans	θ_max_ = 25°, θ_min_ = 1.8°
Absorption correction: multi-scan (*CrysAlis PRO*; Oxford Diffraction, 2009)	*h* = −5→5
*T*_min_ = 0.957, *T*_max_ = 0.990	*k* = −6→6
22131 measured reflections	*l* = −54→54

### Refinement

**Table:**

Refinement on *F*^2^	Primary atom site location: structure-invariant direct methods
Least-squares matrix: full	Secondary atom site location: difference Fourier map
*R*[*F*^2^ > 2σ(*F*^2^)] = 0.044	Hydrogen site location: inferred from neighbouring sites
*wR*(*F*^2^) = 0.097	H-atom parameters constrained
*S* = 1.20	*w* = 1/[σ^2^(*F*_o_^2^) + (0.0248*P*)^2^ + 0.5849*P*] where *P* = (*F*_o_^2^ + 2*F*_c_^2^)/3
1414 reflections	(Δ/σ)_max_ < 0.001
165 parameters	Δρ_max_ = 0.12 e Å^−^^3^
0 restraints	Δρ_min_ = −0.13 e Å^−^^3^

### Special details

**Table:**

Geometry. All esds (except the esd in the dihedral angle between two l.s. planes) are estimated using the full covariance matrix. The cell esds are taken into account individually in the estimation of esds in distances, angles and torsion angles; correlations between esds in cell parameters are only used when they are defined by crystal symmetry. An approximate (isotropic) treatment of cell esds is used for estimating esds involving l.s. planes.
Refinement. Refinement of *F*^2^ against ALL reflections. The weighted *R*-factor wR and goodness of fit *S* are based on *F*^2^, conventional *R*-factors *R* are based on *F*, with *F* set to zero for negative *F*^2^. The threshold expression of *F*^2^ > σ(*F*^2^) is used only for calculating *R*-factors(gt) etc. and is not relevant to the choice of reflections for refinement. *R*-factors based on *F*^2^ are statistically about twice as large as those based on *F*, and *R*- factors based on ALL data will be even larger.

### Fractional atomic coordinates and isotropic or equivalent isotropic displacement parameters (Å^2^)

**Table:**

	*x*	*y*	*z*	*U*_iso_*/*U*_eq_	Occ. (<1)
O1	0.1938 (4)	0.2752 (5)	0.11488 (4)	0.0571 (7)	
N1	0.6250 (4)	0.2075 (4)	0.12991 (4)	0.0354 (5)	
H1N	0.7945	0.2132	0.1251	0.043*	
C1	0.4388 (5)	0.2701 (5)	0.10975 (5)	0.0345 (6)	
C2	0.5509 (5)	0.3297 (5)	0.08026 (5)	0.0340 (6)	
C3	0.4651 (6)	0.5263 (5)	0.06577 (6)	0.0436 (7)	
C4	0.5758 (7)	0.5680 (6)	0.03829 (6)	0.0580 (9)	
H4	0.5241	0.6989	0.0282	0.070*	
C5	0.7593 (7)	0.4208 (7)	0.02570 (6)	0.0644 (10)	
H5	0.8294	0.4532	0.0073	0.077*	
C6	0.8400 (7)	0.2271 (7)	0.03994 (6)	0.0600 (9)	
H6	0.9627	0.1267	0.0313	0.072*	
C7	0.7366 (6)	0.1831 (5)	0.06728 (6)	0.0446 (7)	
H7	0.7924	0.0528	0.0772	0.054*	
C8	0.5574 (5)	0.1322 (4)	0.15873 (5)	0.0323 (6)	
C9	0.6678 (6)	−0.0703 (5)	0.16930 (6)	0.0382 (6)	
C10	0.5923 (6)	−0.1364 (5)	0.19709 (6)	0.0484 (8)	
H10	0.6660	−0.2696	0.2048	0.058*	
C11	0.4121 (6)	−0.0122 (5)	0.21371 (6)	0.0488 (8)	
H11	0.3631	−0.0644	0.2321	0.059*	
C12	0.3027 (6)	0.1906 (5)	0.20322 (5)	0.0399 (7)	
C13	0.3794 (5)	0.2610 (5)	0.17554 (5)	0.0365 (6)	
H13	0.3103	0.3969	0.1681	0.044*	
C14	0.2646 (7)	0.6906 (6)	0.07875 (8)	0.0633 (9)	
H14A	0.2576	0.8264	0.0670	0.095*	
H14B	0.3206	0.7294	0.0982	0.095*	
H14C	0.0872	0.6213	0.0793	0.095*	
C15	0.8619 (6)	−0.2117 (5)	0.15136 (6)	0.0527 (8)	
H15A	1.0349	−0.1354	0.1502	0.079*	
H15B	0.7888	−0.2316	0.1321	0.079*	
H15C	0.8857	−0.3583	0.1604	0.079*	
C16	0.1035 (7)	0.3287 (6)	0.22091 (6)	0.0566 (9)	
H16A	0.1199	0.2888	0.2412	0.068*	0.74
H16B	−0.0786	0.2971	0.2144	0.068*	0.74
H16C	0.1420	0.4883	0.2185	0.068*	0.74
H16D	0.0025	0.4276	0.2082	0.068*	0.26
H16E	0.2007	0.4188	0.2350	0.068*	0.26
H16F	−0.0201	0.2279	0.2309	0.068*	0.26

### Atomic displacement parameters (Å^2^)

**Table:**

	*U*^11^	*U*^22^	*U*^33^	*U*^12^	*U*^13^	*U*^23^
O1	0.0242 (10)	0.0968 (18)	0.0502 (11)	0.0001 (13)	0.0046 (9)	0.0132 (13)
N1	0.0211 (10)	0.0470 (13)	0.0382 (11)	0.0021 (11)	0.0061 (9)	0.0045 (11)
C1	0.0267 (13)	0.0385 (15)	0.0382 (14)	−0.0019 (13)	0.0028 (11)	−0.0007 (13)
C2	0.0242 (12)	0.0429 (15)	0.0349 (13)	−0.0036 (13)	−0.0003 (11)	−0.0024 (12)
C3	0.0354 (15)	0.0501 (17)	0.0454 (16)	−0.0044 (15)	−0.0066 (13)	0.0029 (14)
C4	0.058 (2)	0.068 (2)	0.0477 (17)	−0.005 (2)	−0.0123 (17)	0.0192 (17)
C5	0.059 (2)	0.100 (3)	0.0339 (15)	−0.011 (2)	0.0050 (15)	0.0039 (19)
C6	0.0532 (19)	0.084 (2)	0.0424 (16)	0.005 (2)	0.0094 (15)	−0.0104 (18)
C7	0.0376 (15)	0.0532 (18)	0.0431 (15)	0.0033 (16)	0.0023 (13)	−0.0010 (14)
C8	0.0271 (13)	0.0359 (14)	0.0340 (13)	−0.0028 (13)	0.0029 (12)	0.0016 (11)
C9	0.0315 (14)	0.0402 (15)	0.0429 (15)	−0.0003 (14)	0.0006 (12)	0.0004 (12)
C10	0.0488 (17)	0.0452 (17)	0.0511 (17)	0.0070 (17)	0.0016 (15)	0.0145 (14)
C11	0.0489 (18)	0.0584 (19)	0.0392 (15)	0.0001 (18)	0.0067 (15)	0.0120 (15)
C12	0.0337 (14)	0.0497 (17)	0.0363 (13)	−0.0020 (15)	0.0046 (12)	−0.0020 (13)
C13	0.0331 (13)	0.0369 (14)	0.0395 (14)	0.0044 (14)	0.0022 (11)	0.0016 (12)
C14	0.0526 (19)	0.0535 (19)	0.084 (2)	0.0100 (19)	−0.0045 (18)	0.0053 (19)
C15	0.0522 (18)	0.0435 (17)	0.0625 (18)	0.0149 (18)	0.0056 (15)	0.0020 (15)
C16	0.0529 (19)	0.072 (2)	0.0449 (16)	0.007 (2)	0.0120 (15)	−0.0044 (16)

### Geometric parameters (Å, °)

**Table:**

O1—C1	1.224 (3)	C10—C11	1.375 (4)
N1—C1	1.349 (3)	C10—H10	0.9300
N1—C8	1.431 (3)	C11—C12	1.389 (4)
N1—H1N	0.8598	C11—H11	0.9300
C1—C2	1.500 (3)	C12—C13	1.386 (3)
C2—C7	1.386 (4)	C12—C16	1.505 (4)
C2—C3	1.394 (4)	C13—H13	0.9300
C3—C4	1.393 (4)	C14—H14A	0.9600
C3—C14	1.499 (4)	C14—H14B	0.9600
C4—C5	1.373 (5)	C14—H14C	0.9600
C4—H4	0.9300	C15—H15A	0.9600
C5—C6	1.367 (5)	C15—H15B	0.9600
C5—H5	0.9300	C15—H15C	0.9600
C6—C7	1.376 (4)	C16—H16A	0.9599
C6—H6	0.9300	C16—H16B	0.9598
C7—H7	0.9300	C16—H16C	0.9602
C8—C13	1.387 (3)	C16—H16D	0.9605
C8—C9	1.391 (4)	C16—H16E	0.9588
C9—C10	1.381 (4)	C16—H16F	0.9607
C9—C15	1.505 (4)		
			
C1—N1—C8	124.0 (2)	C11—C12—C16	121.6 (3)
C1—N1—H1N	117.9	C12—C13—C8	121.2 (3)
C8—N1—H1N	118.1	C12—C13—H13	119.4
O1—C1—N1	122.6 (2)	C8—C13—H13	119.4
O1—C1—C2	121.8 (2)	C3—C14—H14A	109.5
N1—C1—C2	115.6 (2)	C3—C14—H14B	109.5
C7—C2—C3	120.4 (3)	H14A—C14—H14B	109.5
C7—C2—C1	118.9 (2)	C3—C14—H14C	109.5
C3—C2—C1	120.7 (2)	H14A—C14—H14C	109.5
C4—C3—C2	117.3 (3)	H14B—C14—H14C	109.5
C4—C3—C14	120.1 (3)	C9—C15—H15A	109.5
C2—C3—C14	122.6 (3)	C9—C15—H15B	109.5
C5—C4—C3	121.7 (3)	H15A—C15—H15B	109.5
C5—C4—H4	119.2	C9—C15—H15C	109.5
C3—C4—H4	119.2	H15A—C15—H15C	109.5
C6—C5—C4	120.7 (3)	H15B—C15—H15C	109.5
C6—C5—H5	119.7	C12—C16—H16A	109.6
C4—C5—H5	119.7	C12—C16—H16B	109.3
C5—C6—C7	118.9 (3)	H16A—C16—H16B	109.5
C5—C6—H6	120.5	C12—C16—H16C	109.4
C7—C6—H6	120.5	H16A—C16—H16C	109.5
C6—C7—C2	121.1 (3)	H16B—C16—H16C	109.5
C6—C7—H7	119.4	C12—C16—H16D	109.3
C2—C7—H7	119.4	H16A—C16—H16D	141.1
C13—C8—C9	121.0 (2)	H16B—C16—H16D	56.4
C13—C8—N1	119.4 (2)	H16C—C16—H16D	56.1
C9—C8—N1	119.6 (2)	C12—C16—H16E	109.6
C10—C9—C8	117.1 (3)	H16A—C16—H16E	56.2
C10—C9—C15	121.2 (3)	H16B—C16—H16E	141.1
C8—C9—C15	121.6 (2)	H16C—C16—H16E	56.3
C11—C10—C9	122.3 (3)	H16D—C16—H16E	109.4
C11—C10—H10	118.8	C12—C16—H16F	109.5
C9—C10—H10	118.8	H16A—C16—H16F	56.3
C10—C11—C12	120.6 (3)	H16B—C16—H16F	56.2
C10—C11—H11	119.7	H16C—C16—H16F	141.1
C12—C11—H11	119.7	H16D—C16—H16F	109.5
C13—C12—C11	117.8 (3)	H16E—C16—H16F	109.5
C13—C12—C16	120.6 (3)		
			
C8—N1—C1—O1	2.7 (5)	C1—C2—C7—C6	178.3 (3)
C8—N1—C1—C2	−176.3 (2)	C1—N1—C8—C13	−49.7 (4)
O1—C1—C2—C7	−130.9 (3)	C1—N1—C8—C9	129.3 (3)
N1—C1—C2—C7	48.1 (3)	C13—C8—C9—C10	0.0 (4)
O1—C1—C2—C3	47.5 (4)	N1—C8—C9—C10	−179.1 (3)
N1—C1—C2—C3	−133.5 (3)	C13—C8—C9—C15	−179.8 (3)
C7—C2—C3—C4	−0.7 (4)	N1—C8—C9—C15	1.1 (4)
C1—C2—C3—C4	−179.1 (3)	C8—C9—C10—C11	1.3 (4)
C7—C2—C3—C14	180.0 (3)	C15—C9—C10—C11	−178.9 (3)
C1—C2—C3—C14	1.5 (4)	C9—C10—C11—C12	−1.6 (5)
C2—C3—C4—C5	0.8 (4)	C10—C11—C12—C13	0.6 (4)
C14—C3—C4—C5	−179.8 (3)	C10—C11—C12—C16	179.5 (3)
C3—C4—C5—C6	−0.1 (5)	C11—C12—C13—C8	0.6 (4)
C4—C5—C6—C7	−0.7 (5)	C16—C12—C13—C8	−178.3 (2)
C5—C6—C7—C2	0.9 (5)	C9—C8—C13—C12	−0.9 (4)
C3—C2—C7—C6	−0.2 (4)	N1—C8—C13—C12	178.1 (2)

### Hydrogen-bond geometry (Å, °)

**Table:**

*D*—H···*A*	*D*—H	H···*A*	*D*···*A*	*D*—H···*A*
N1—H1N···O1^i^	0.86	2.05	2.899 (3)	172

Symmetry codes: (i) *x*+1, *y*, *z*.
